# Association of *EPHX2* R287Q Polymorphism with Diabetic Nephropathy in Chinese Type 2 Diabetic Patients

**DOI:** 10.1155/2018/2786470

**Published:** 2018-02-05

**Authors:** Liang Ma, Meihua Yan, Xiaomu Kong, Yongwei Jiang, Tingting Zhao, Hailing Zhao, Qian Liu, Haojun Zhang, Peng Liu, Yongtong Cao, Ping Li

**Affiliations:** ^1^Clinical Laboratory, China-Japan Friendship Hospital, Beijing, China; ^2^Beijing Key Lab Immune-Mediated Inflammatory Diseases, Institute of Clinical Medical Science, China-Japan Friendship Hospital, Beijing, China; ^3^Department of Endocrinology, China-Japan Friendship Hospital, Beijing, China; ^4^Graduate School of Peking Union Medical College, Chinese Academy of Medical Sciences and Peking Union Medical College, Beijing, China

## Abstract

The aim of this study was to investigate the relationship between *EPHX2* rs751141 (R287Q polymorphism) and diabetic nephropathy (DN) in Chinese type 2 diabetes (T2D). This case-control study explored the association between *EPHX2* rs751141 and DN in a total of 870 Chinese T2D patients (406 T2D patients with DN and 464 T2D patients without DN). DNA was extracted from peripheral leukocytes of the patients and rs751141 was genotyped. The A allele frequency of rs751141 was significantly lower in DN patients (20.94%) compared with non-DN controls (27.8%) (*P* = 0.001), and the A allele of rs751141 was associated with a significantly lower risk of DN after adjustment for multiple covariates in the additive genetic model (OR = 0.68, 95% CI = 0.52–0.88, *P* = 0.004). Significant association between rs751141 and homocysteine (Hcy) level on the risk of DN was observed, indicating that in patients with the highest Hcy levels, the A allele showed marked association with lower risk of DN in all three genetic models. In conclusion, the A allele of exonic polymorphism in EPHX2 rs751141 is negatively associated with the incidence of DN in the Chinese T2D population, which could be modulated by Hcy level status.

## 1. Introduction

In China, as of 2010, there were more than 110 million adults with diabetes mellitus and another more than 490 million with prediabetes [1]. Approximately 30–40% of type 2 diabetes (T2D) patients develop diabetic nephropathy (DN), a common microvascular complication of DM that may eventually require renal replacement therapy [2]. This is a warning sign for healthcare systems to be vigilant not only for adequate management but also for identifying risk factors involved in the pathogenesis of DN. In addition to conventional modifiable risk factors (hypertension, smoking, hyperlipidemia, and hemodynamic changes), hereditary components are also considered to be a significant risk factor for DN. Since DN occurs in familial clusters, genetic factors may contribute to DN development although the incidence and severity of DN are affected by the extent of blood glucose status [3, 4]. Therefore, identifying the genetic factors of DN can be of great significance in the prediction and prevention of DN in T2D in the Chinese population.

Epoxyeicosatrienoic acids (EETs) are involved in regulation of renal blood flow and long-term arterial blood pressure. Moreover, renal and cardiovascular diseases are associated with decreased renal and vascular levels of EETs [5]. In different animal models, high levels of EETs have been shown to protect kidney injury through anti-inflammatory, antiplatelet, and antiproliferative activities and endothelial-derived hyperpolarizing role [6–8]. However, EETs are degraded to corresponding diols by a soluble epoxide hydrolase (sEH) that is encoded by the *EPHX2* gene on chromosome 8p21-p12 [9]. Reducing sEH activity by a small molecular inhibitor can increase EET levels and decrease their degradation [10–12]. Gene knockout of *EPHX2* can improve endothelial function and reduce renal injury in streptozotocin-induced diabetic mice [13]. Furthermore, *EPHX2* deletion attenuates renal injury and inflammation in DOCA-salt hypertension [14]. Several single nucleotide polymorphisms (SNPs) of *EPHX2* have been identified, and one of the known SNPs is R287Q polymorphism (rs751141). The presence of an A instead of a G allele results in a lower sEH activity of approximately 25–58% [15].

Although the relationship between rs751141 and risk of IgA nephropathy has been investigated [16], patients carrying the A allele of rs751141 possessed better kidney survival than those with the G allele. However, the association between rs751141 and DN is unknown. Accordingly, we tested the hypothesis that an association exists between *EPHX2* rs751141 and risk of DN in the Chinese with T2D and whether Hcy level affects this association.

## 2. Materials and Methods

This study was approved by the institutional ethics committee of the China-Japan Friendship Hospital, Beijing, China. Signed informed consent was obtained from all participants. A total of 870 Chinese T2D patients were recruited from the China-Japan Friendship Hospital, between February 2015 and June 2016.

T2D was defined by the World Health Organization (WHO) 1999 criteria. This was a clinic-based case-control study. Cases were persons with type 2 diabetes and DN, and controls were persons with type 2 diabetes who did not have DN. Inclusion criteria for cases were diagnosed with T2D, age between 35 and 85 years old, and 24 h urinary albumin > 500 mg/L or an albumin creatinine ratio (ACR) > 30 mg/g. Inclusion criteria for controls were T2D duration ≥ 7 years, age between 35 and 85 years old, and 24 h urinary albumin < 150 mg/L or an ACR < 30 mg/g. Exclusion criteria for both groups consisted of known proteinuria before the onset of diabetes, other primary or secondary renal diseases (e.g., IgA nephropathy, membranous nephropathy, lupus nephritis, obstructive renal disease, renal stone disease, and acute urinary tract infection), and malignancy.

### 2.1. Data Collection

Demographic information, smoking habit, history of hypertension, body mass index (BMI), systolic blood pressure (SBP), diastolic blood pressure (DBP), 24 h urinary albumin excretion and ACR, high-density lipoprotein cholesterol (HDL-C), low-density lipoprotein cholesterol (LDL-C), total cholesterol (TC), triglycerides (TG), and homocysteine (Hcy) of each participant were obtained. Body weight and height were measured using standard methods, and BMI was calculated as weight (kg) divided by height squared (m^2^). Resting blood pressure was measured twice according to standard protocol and the results were averaged. Serum concentrations of fasting TG, TC, LDL-C, HDL-C, and Hcy were measured using an automated biochemical analyzer (AU5800 Clinical Chemistry System, Beckman Coulter, Brea, CA, USA). A1C was measured using the D-10 Hemoglobin Testing System (Bio-Rad, Hercules, CA, USA).

### 2.2. DNA Extraction

DNA was extracted from peripheral blood using the QIAamp DNA Blood Mini Kit (Qiagen, Hilden, Germany) following the manufacturer's recommendations and then stored at −20°C or amplified immediately. The concentration of DNA was determined using the NanoDrop 1000 spectrophotometer (Thermo Scientific, Waltham, MA, USA).

### 2.3. Amplification and Detection of *EPHX2* Gene R287Q Polymorphism

Genotyping was confirmed using TaqMan SNP Genotyping Assay (Applied Biosystems, Waltham, MA, USA). In all, 50 ng DNA was amplified in a 25 *μ*l reaction mixture containing 12.5 *μ*l of Premix Ex Taq (Probe qPCR) (Takara, Japan), 5 pmol of each primer (Applied Biosystems) and 3 pmol of each probe (Applied Biosystems) for the amplification of *EPHX2*. The primer and probe sequences were custom designed and synthesized by Applied Biosystems. The primer sequences were F:5′-CGGGAGGAGCAGATGACTCT-3′ and R:5′-TGGAGTGTGCCTGTTTGTTTTC-3′. The probe sequences were FAM-5′-CATAGCTAGGACCCGGTAACCTGCCT-3′-TAMRA and 5′VIC-5′-CCATAGCTAGGACCTGGTAACCTGCCT-3′-TAMRA.

Amplification was performed using a real-time polymerase chain reaction (PCR) detector (LightCycler 480, Roche Diagnostics, Penzberg, Germany) with a PCR temperature profile consisting of denaturation at 95°C for 10 minutes following 40 cycles of denaturation at 95°C for 15 seconds, annealing and elongation at 65°C for 60 seconds.

### 2.4. Genotyping Using DNA Sequencing

To confirm the genotyping results, 50 samples were randomly selected for DNA sequencing. rs751141 was amplified for DNA sequencing using the following designed primers F:5′-TTACAGGAAGAAGGGGATGG-3′ and R:5′-GGCAGGTAGAAGGCAAGACC-3′. Amplifications followed standard protocol, and the PCR products were purified using the QIAquick PCR Purification Kit (Qiagen) and subsequently analyzed by direct sequencing with an automated DNA sequencer (3500 Genetic Analyzer, Applied Biosystems).

### 2.5. Statistical Analysis

Quantitative clinical data (age, BMI, blood pressure, duration of diabetes, total cholesterol, HDL-C, LDL-C, TG, and Hcy) were non-Gaussian distribution and presented as median (interquartile range), and Wilcoxon's test was used to compare the difference in clinical characteristics between DN and DM groups. Genotype distribution and allelic frequency were analyzed using the chi-square test. Deviations from Hardy-Weinberg were also tested using the chi-square test. Finally, multiple logistic regression analyses were carried out to examine the association between rs751141 and risk of DN adjusted for age, sex, duration of diabetes, history of hypertension, smoking status, cholesterol, TG, and Hcy levels in additive, recessive, or dominant models. To define these models, take SNP rs751141 as an example where A is the minor allele. For the dominant model, AA and GA were coded as 1 in the regression model and GG was coded 0. For the recessive model, AA was coded as 1 while GG and GA were coded as 0. For the additive model, AA, GA, and GG were coded as 2, 1, and 0, respectively.

Power calculation was performed by Quanto software (version 1.2.4, University of Southern California, Los Angeles, CA, USA). Data were analyzed with the SPSS software (version 17.0, IBM, Armonk, NY, USA). *P* values of < 0.05 were considered significant.

## 3. Results

### 3.1. Baseline Characteristics

A total of 870 participants were included in this study. Cases included 406 DM patients with DN patients (258 males and 148 females), and controls included 464 DM patients without DN (271 males and 191 females) (Table 1). Variables such as smoking, BMI, blood pressure, HDL-C, history of hypertension, TG, and Hcy were found to be elevated in DN patients compared to controls (Table 1).

### 3.2. Distribution of *EPHX2* R287Q Genotype

Distribution of allele frequencies of rs751141 was in accordance with the Hardy-Weinberg equilibrium in both DM with DN and without DN participants (*P* = 0.683 and *P* = 0.354, resp.). Genetic distribution of rs751141 (GG, GA, and AA) was found to be significantly different between DM participants with and without DN (*P* = 0.001) as well as allele frequencies (A and G alleles) (*P* = 0.001) (Table 2).

### 3.3. Association of R287Q Polymorphism with DN Risk

To confirm the association between the genotyping of rs751141 and the possibility of DN, further multiple logistic regression analysis was performed. Three statistical models were used as unadjusted, adjusted for age and sex, and adjusted for age, sex, Hcy, BMI, duration of diabetes, hypertension, smoking, total cholesterol, and TG levels. Results indicated the risk of DN with rs751141 in these models (Table 3). Additive and recessive models were significantly different in participants with DM patients with DN and DM patients without DN. Furthermore, the dominant model displayed a tendency toward significant difference (*P* = 0.059). AA genotyping was found to significantly decrease the risk of DN compared with GG genotyping in unadjusted and adjusted models (*P* < 0.001).

### 3.4. R287Q Genotype and Hcy Level Interaction

Participants were randomly divided into three equal groups based on Hcy levels (low level: 4.29–10.78 umol/L; medium level: 10.79–13.93 umol/L; high level: 13.94–55.99 umol/L). The three genetic models of additive, recessive, and dominant showed significant association with DN in the high level of Hcy groups in unadjusted (*P* = 0.005, *P* = 0.02, and *P* = 0.02, resp.) and adjusted models (*P* = 0.004, *P* = 0.015, and *P* = 0.019, resp.), indicating that the A allele carriers in the high Hcy group were at lower risk of DN, while no significant association was observed in the medium (*P* = 0.38, *P* = 0.10, and *P* = 0.81, resp.) and low Hcy groups (*P* = 0.113, *P* = 0.05, and *P* = 0.35, resp.) in the adjusted model (Table 4).

## 4. Discussion

To our knowledge, this study is the first to describe the association between the polymorphism rs751141 of *EPHX2* and DN, as well as the influence of Hcy levels status on their association. A significant difference in the genotypic distribution of rs751141 between DM with DN and without DN participants was observed (*P* = 0.001), and the frequency of the A allele of rs751141 was significantly higher in the DM without the DN group than in the DM with DN group (*P* = 0.001). This suggests that the presence of the A allele of rs751141 was associated with a protective factor for against DN. This finding is consistent with the hypothesis that decreased endogenous sEH metabolic activity increases EET level, such that A allele carriers were associated with a high level of EET and lower risk of DN compared with individuals with GG genotype after adjustment for sex, age, smoking, BMI, history of hypertension, Hcy level, duration of diabetes, TG, and total cholesterol in the current investigation under the additive and recessive genetic models and that a similar trend was observed under the dominant genetic model (*P* = 0.059). Compared with the GG genotype, the AA genotype was found to confer 73% decreased the risk of DN after adjusting for established DN risk factors. The A allele of rs751141 appears to be an independent protective factor against DN in Chinese T2D patients. Moreover, a previous study found that there was no significant difference in genotype distribution and allele frequency between nondiabetic persons and T2D patients [17].

Studies have demonstrated that the A allele of rs751141 exhibits markedly lower sEH metabolic activity and decreased EET hydrolysis in vitro [15, 18]. sEH is an enzyme with multiple biological functions and is involved in the metabolism of xenobiotics. EETs are the endogenous substrates of sEH [19]. The decreased production of 20-hydroxyeicosatetraenoic acid (20-HETE) and EETs in kidney glomeruli is an important factor in glomerular damage in the early stage of DN [20]. In contrast, studies have shown that increase in EETs exerts renal protective effects and 8-9 EET may have a unique and specific protective role in the glomerular filtration barrier [21]. Levels of EETs depend not only on hydrolysis to dihydroxyeicosatrienoic acids (DHETs) by sEH but also on their production by cytochromes P450 (CYPs). Endothelial CYP2J2 overexpression can metabolize arachidonic acid into EETs, which protects the kidney against development and progression of chronic failure [22]. Previous studies have suggested that endothelial dysfunction is a universal risk factor of cardiovascular and renal diseases [23, 24]. Our findings also support the results of a previous study that the A allele of rs751141 is associated with a significantly lower risk of ischemic stroke in a Chinese population [25, 26], suggesting a protective role of the A allele of rs751141 in type 2 DN.

Interactions between genetic and environmental factors play a substantial role in disease risk [27]. A high Hcy level is considered a major risk factor of DN and accelerates disease progression. In the present study, we also observed the effect of high Hcy level on the association between rs751141 and DN in three genetic models. High Hcy level can downregulate CYP2J2 protein expression by inducing matrix metallopeptidase 9 (MMP-9) activation [28] and upregulate sEH protein expression through endoplasmic reticulum stress, activation of activating transcription factor-6 (ATF6), and DNA methylation in vitro and in vivo [29]. Increased sEH protein expression plays an important role in Hcy-induced endothelial activation, which can be prevented by inhibition of enzyme activity [29]. Levels of EETs depend not only on hydrolysis to dihydroxyeicosatrienoic acids (DHETs) by sEH but also on their production by CYPs. Therefore, decreased CYP2J2 expression and increased sEH expression likely attenuate the protective effect by reducing the production of EETs. Hcy level stratification indicated that a high Hcy level has an important genetic effect on rs751141 on the risk of DN. The A allele showed a protective effect in the high Hcy level group but not in the medium and low Hcy groups. This indicates that interactions between genetic and environmental risk factors may be important for the development of DN. Future studies in a larger population are required to better characterize this potential gene-environment interaction and the mechanistic contribution of *EPHX2* and Hcy to DN risk in persons with T2D.

The present study was performed with 406 patients with DN and 464 control participants with DM, which would have good statistical power to detect associations. However, there are still some limitations. First, the sample size of this study was limited when stratified according to Hcy level. Second, this study was conducted in Chinese and whether the results can be generalized to other ethnic groups need further investigation. Thus, a more extensive examination across various ethnic groups may help clarify the role of *EPHX2* rs751141 in the etiology of DN. Finally, the precise biological mechanism of the protective effect of the A allele of rs751141 against DN needs further elucidation.

In conclusion, our findings revealed that the exonic genetic variant in *EPHX2* rs751141 is significantly associated with the development of type 2 DN in the Chinese patients, suggesting the essential role that sEH plays in the pathogenesis of DN. In addition, Hcy level status affected the genetic risk for type 2 DN. Pharmacologic inhibition of sEH is being investigated as a novel therapeutic strategy for CKD [30, 31]. Therefore, based on our observation, sEH inhibitors may be used selectively to decrease DN risk in carriers of individuals with high genetic risk.

## Figures and Tables

**Table 1:** Characteristics of DM patients with DN (cases) and without DN (controls).

	DM patients with DN (*n* = 406)^a^	DM patients without DN (*n* = 464)^a^	*P*
Age (years)	63 (54, 71)	61 (54, 68)	0.001
Sex, male (%)	63.55 (258/406)	58.41 (271/464)	0.121
BMI (kg/m^2^)	25.80 (23.89, 28.36)	25.36 (23.20, 27.68)	0.006
Duration of diabetes (years)	15 (9, 21)	13 (10, 18)	0.097
History of hypertension (%)	78.82 (320/406)	49.35 (229/464)	<0.001
Smoking (%)	34.73 (141/406)	28.23 (131/464)	0.039
SBP (mmHg)	138 (125, 150)	127 (120, 140)	<0.001
DBP (mmHg)	80 (74, 84)	80 (70, 80)	0.048
A1C (%)	7.6 (6.5, 9.3)	7.9 (6.7,9.3)	0.148
Hcy (umol/L)	13.63 (11.03, 17.12)	11.48 (9.51, 13.42)	<0.001
TC (mmol/L)	4.25 (3.44, 5.06)	4.14 (3.53, 4.86)	0.36
HDL-C (mmol/L)	0.96 (0.78, 1.18)	1.02 (0.85, 1.24)	0.004
LDL-C (mmol/L)	2.39 (1.85, 2.98)	2.38 (1.94, 2.97)	0.535
TG (mmol/L)	1.71 (1.20, 2.56)	1.42 (0.99, 2.18)	<0.001

BMI: body mass index; DBP: diastolic blood pressure; Hcy: homocysteine; HDL-C: high-density lipoprotein cholesterol; LDL-C: low-density lipoprotein cholesterol SBP: systolic blood pressure; TC: triglyceride. ^a^Data are shown as median (interquantile range) or %.

**Table 2:** Genotype distribution and allele frequency of rs751141 in DM patients with DN and without DN participants.

Genotype	Genotype frequencies		*P*
	DM patients with DN	DM patients without DN	
GG	250	251	0.001
GA	142	168	
AA	14	45	
A allele	20.94%	27.8%	0.001
G allele	79.06%	72.2%	

DM: diabetes mellitus; DN: diabetic nephropathy.

**Table 3:** Odds ratios and 95% confidence interval for DN under three genetic models.

Genetic models	Unadjusted		Adjusted^a^		Adjusted^b^	
	OR (95% CI)	*P*	OR (95% CI)	*P*	OR (95% CI)	*P*
Additive	0.69 (0.55–0.86)	<0.001	0.66 (0.52–0.83)	<0.001	0.68 (0.52–0.88)	0.004
Recessive	0.33 (0.18–0.62)	<0.001	0.33 (0.17–0.65)	0.001	0.28 (0.14–0.60)	0.001
Dominant	0.72 (0.55–0.95)	0.018	0.67 (0.50–0.90)	0.008	0.73 (0.53–1.01)	0.059
GA versus GG	0.83 (0.63–1.10)	0.201	0.77 (0.57–1.05)	0.097	0.87 (0.62–1.23)	0.43
AA versus GG	0.31 (0.17–0.58)	<0.001	0.30 (0.15–0.60)	<0.001	0.27 (0.13–0.58)	<0.001
Allele (A versus G)	0.69 (0.55–0.86)	<0.001				

CI: confidence interval; DN: diabetic nephropathy; OR: odds ratio. ^a^Adjusted for age and sex. ^b^Adjusted for age, sex, Hcy, BMI, duration of diabetes, hypertension, smoking, total cholesterol, and TG levels.

**Table 4:** Association of R287Q with risk of DN in different Hcy-level groups.

Hcy level (umol/L)		Unadjusted		Adjusted^a^	
	Genotype	OR (95% CI)	*P*	OR (95% CI)	*P*
Low: 4.29–10.78 (*n* = 290)	Additive	0.67 (0.44–1.02)	0.063	0.67 (0.41–1.10)	0.113
	Recessive	0.42 (0.14–1.27)	0.12	0.18 (0.03–0.98)	0.05
	Dominant	0.65 (0.38–1.11)	0.12	0.74 (0.40–1.38)	0.35
Medium:10.79–13.93 (*n* = 290)	Additive	0.74 (0.49–1.11)	0.14	0.81 (0.51–1.30)	0.38
	Recessive	0.22 (0.05–0.98)	0.047	0.27 (0.06–1.28)	0.10
	Dominant	0.82 (0.51–1.32)	0.41	0.93 (0.53–1.65)	0.81
High:13.94–55.99 (*n* = 290)	Additive	0.57 (0.38–0.84)	0.005	0.52 (0.33–0.81)	0.004
	Recessive	0.32 (0.13–0.82)	0.02	0.27 (0.09–0.78)	0.015
	Dominant	0.56 (0.34–0.92)	0.02	0.51 (0.29–0.90)	0.019

DN: diabetic nephropathy; Hcy: homocysteine. ^a^Adjusted for age, sex, BMI, duration of diabetes, hypertension, smoking, total cholesterol, and TG levels.
